# Supplementary figures and images for: Prevalence and distribution pattern of malaria and soil-transmitted helminth co-endemicity in sub-Saharan Africa, 2000–2018: A geospatial analysis
Source: PLoS Negl Trop Dis. 2022 Sep 30;16(9):e0010321. doi: 10.1371/journal.pntd.0010321 (PMC9555675; doi:10.1371/journal.pntd.0010321)

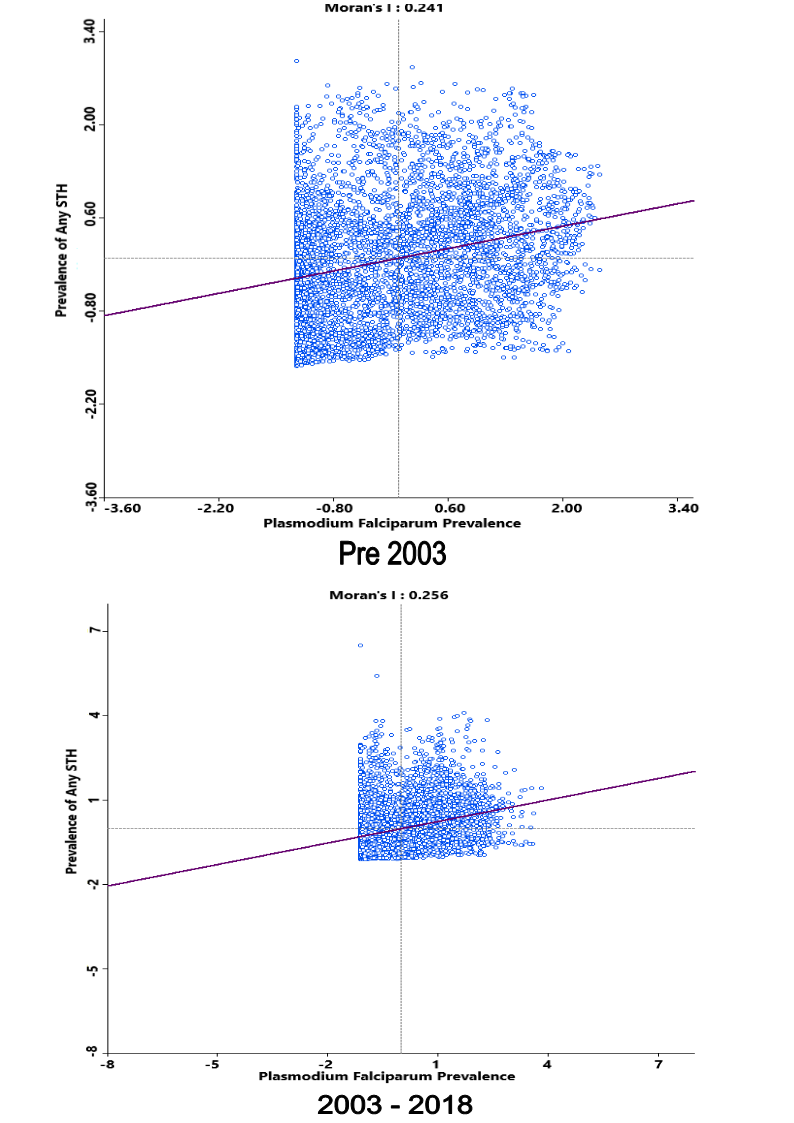

Supplement: S1 Fig — (TIF) [file pntd.0010321.s001.tif]

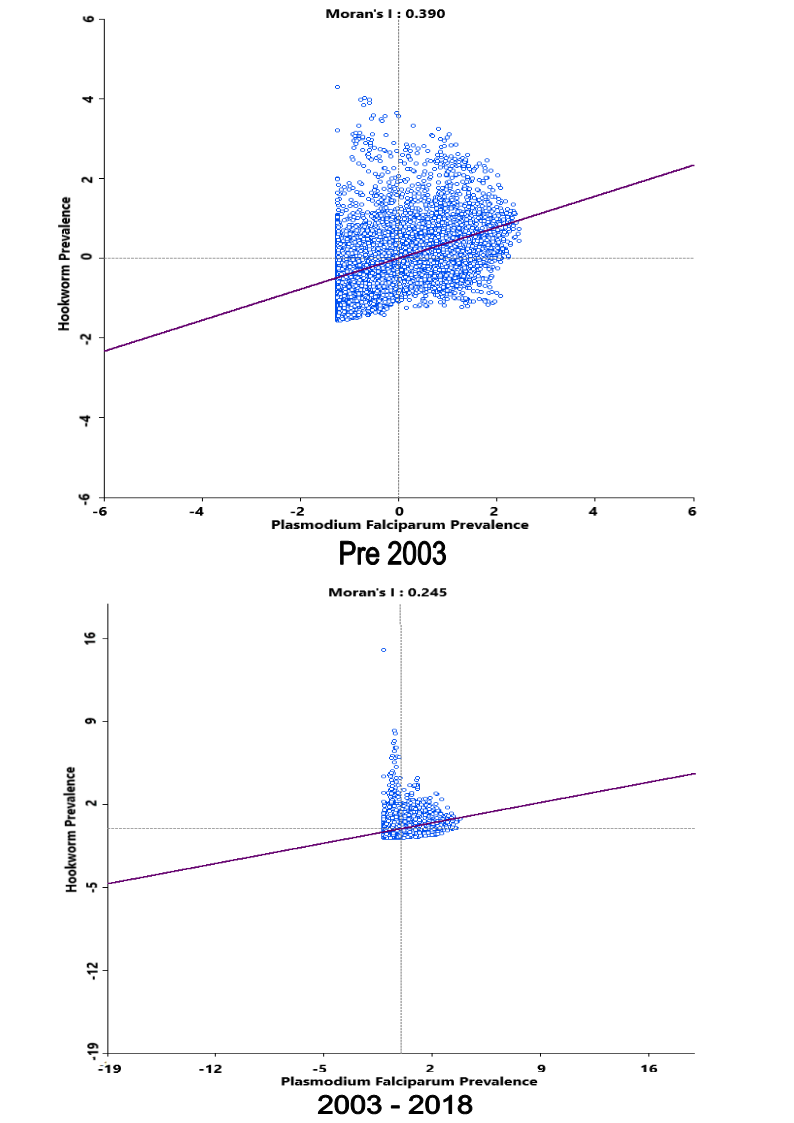

Supplement: S2 Fig — (TIF) [file pntd.0010321.s002.tif]

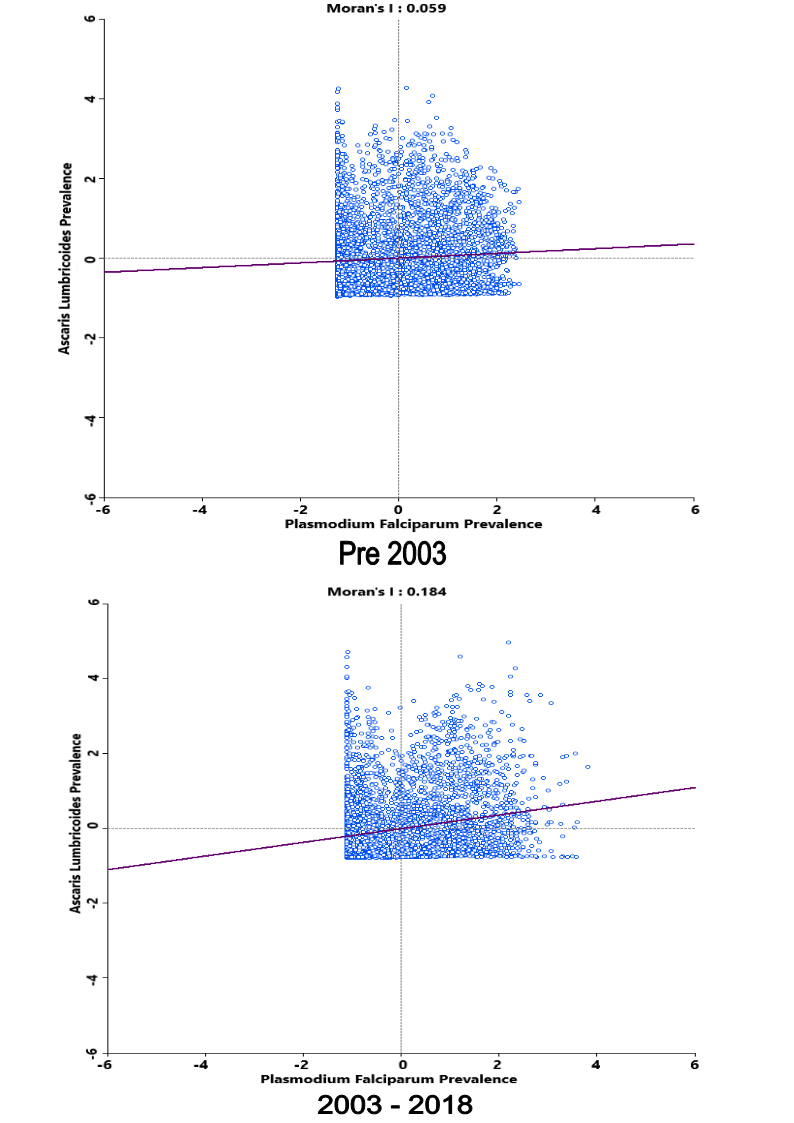

Supplement: S3 Fig — (TIF) [file pntd.0010321.s003.tif]

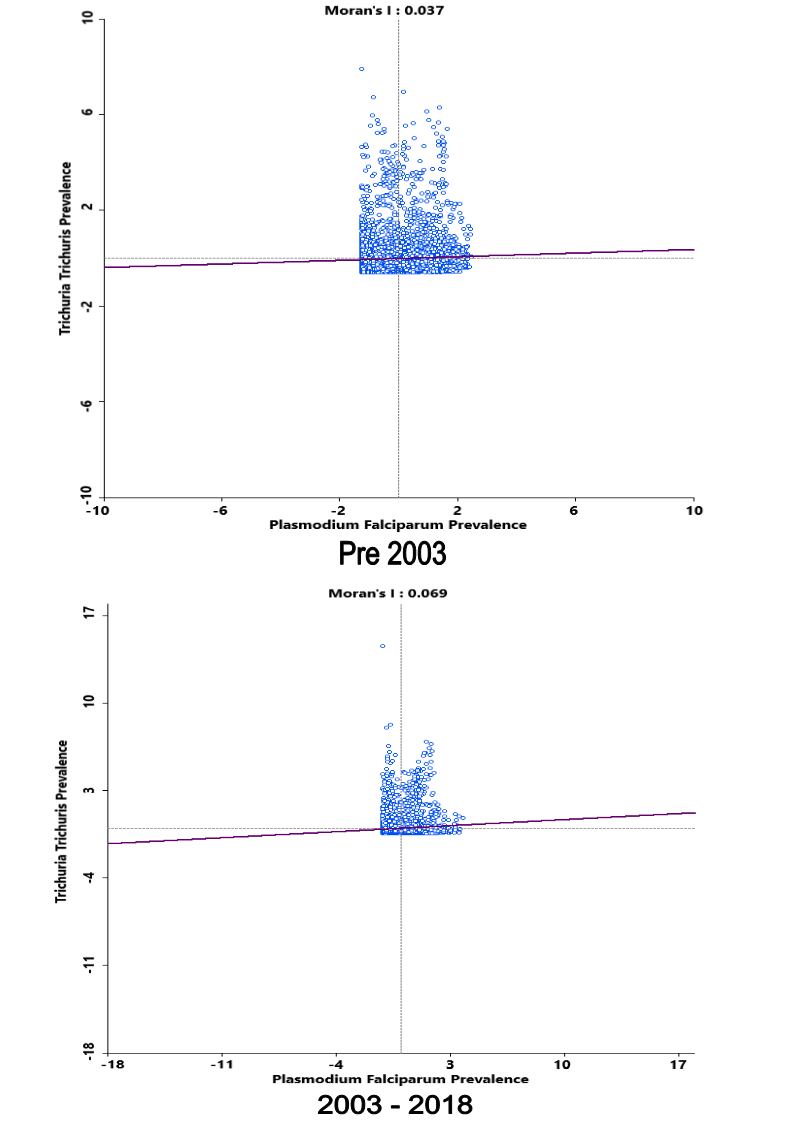

Supplement: S4 Fig — (TIF) [file pntd.0010321.s004.tif]
