# Supplementary material for: Prevalence and distribution pattern of malaria and soil-transmitted helminth co-endemicity in sub-Saharan Africa, 2000–2018: A geospatial analysis
Source: PLoS Negl Trop Dis. 2022 Sep 30;16(9):e0010321. doi: 10.1371/journal.pntd.0010321 (PMC9555675; doi:10.1371/journal.pntd.0010321)
Supplement: S1 Table — (DOCX) [file pntd.0010321.s005.docx]

**S1 Table:** The prevalence of *P. falciparum* and soil-transmitted helminth (Hookworm, *Ascaris lumbricoides, Trichiuris trichiura*) co-infection, and their corresponding significant hotspots locations (ADM1, ADM2, and ADM3) in sub-Saharan Africa, pre-2003 and 2003-2018.

| ***Plasmodium falciparum* and any soil transmitted helminth co-infection** | | | | |
| --- | --- | --- | --- | --- |
|  | **Pre 2003 (ADM1=Countries)** | **2003-2018 (ADM1=Countries)** | **Pre 2003 (ADM2/ADM3)** | **2003-2018 (ADM2/ADM3)** |
| **High-high** prevalence of *P. falciparum* and any soil-transmitted helminth. | Benin, Cote d'Ivoire, Sierra Leone, Liberia, Guinea, Togo, Nigeria, Cameroon, Central Africa Republic, Equatorial Guinea, Gabon, Congo, Angola, Tanzania, Democratic Republic of the Congo, Burundi, Uganda, Zambia, Mozambique, Madagascar | Benin, Cote d'Ivoire, Guinea, Liberia, Nigeria, DRC, Cameroon, Equatorial Guinea, Gabon, Congo, Angola, Mozambique. | **Benin:** Zou and Kouffo regions, Bopa district in the Mono region, Bassila district in the Donga region, Toffo, Allada and Ze district in the Atlantique region, Bonou district in the Oueme region, Adja-Ouere and Ketou district in the Plateau region. The entire Collines region except the Ouesse district.  **Cote d’Ivoire:**Tabou district in the Bassandra region.  **Liberia:** GrandKru, Sinoe, Maryland, River Gee, Grand Gedeh, Gbapolu, Bong, Grand Cape Mount, Bomi, Montserrado, Margibi regions.  **Sierra Leon**: Moyamba and Bo district in the Southern region.  **Guinea**: Boke district in the Boke region  **Togo**: Yoto district in the Maritime region,the Kara region, Bassar, Kozah and Assoli district in Ogou, Wawa, Kloto, Amou and Haho, Central region.  **Nigeria**: The entire Bayelsa, Akwa Ibom, Rivers and Cross river state, Kwande LGA in Benue State, The whole of Abia state with the exception of Ohafia, Umu-Nneochi, Umuahia, Aba and Isikwuato LGAs, Ussa and Kurmi LGAs in Taraba State, Ohaji, Oguta, Owerri, Ikeduru, Aboh and Ngor areas of Imo State,Burutu, Bomadi, Ndokwa, Warri and Aniocha LGAs of Delta State, Ovia and Orhionmw LGAs in Edo State, Irepodun, Ekiti South-West, Ekiti West,Ikere, Ado and Ise-Orun LGAs in Ekiti State, the whole of Ondo State except Okitipupa and Akoko parts of the State, the whole of Osun State except Orolu, Irepodun, Ifelodun, Boripe, Ila and Ifedayo LGAs, Oyo,Atiba, Afijio, Atisbo, Surulere, Ibadan, Iseyin, Iwajowa, Itesiwaju, Saki, Irepo, Olorunsogo, Orelope LGAs in Oyo State, Ijebu, Remo, Obafemi-Owode, Odeda, Abeokuta, Yewa North and Ogun Waterside LGAs in Ogun State.  **Cameroon**: The entire Littoral, Sud-Ouest and Sud region, Donga Metong, Menchum, Boyo, Momo and Ngo Ketunja districts in the Nord-Ouest region, Haut Nkam, Noun, and Nde districts in the Ouest region, All of Centre region except Mbam et Kim district and All of Est region except Kadey district. Most of these areas.  **Equatorial Guinea**: The entire country from Litoral to Kie-Ntem, Wele-Nzas to Centro Sur.  **Gabon**: Wouleu-Ntem, Estuaire, Ogooue et des Lacs district in Moyen-Ogooue, Mvoung district in Ogooue-Ivindo region, Sebe-Brikolo and lekoko district in Haut- Ogooue region.  **Congo**: The entire Sangha, Cuvette, Cuvette Ouest, Plateaux, Likouala regions, Mayama, Ngabe and Ngamaba districts in the pool region, Madingo-Kayes, Kakamoeka and Mvouti districts in the Kouilou region, Kibangou and Divene district in the Niari region.  **Angola**: The entire Cuaza Norte and Uige region, Cacuzo, Malanje Cuaba, Calandula, Massango districts in Malanje region, Ambriz, Nambuangongo, Dande, Icolo e Bengo districts in the Bengo region.Cacolo in Lunda Sul region, Mussende and Quibala in Cuanza Sul, Camacupa and Andulo in Bie region. All of the Zaire region with the exception of Soyo district. Maquela do Zombo, Damba, Buengas, Bembe, Quitexe and Puri districts in the Uige region and Bolongongo district in Cuaza Norte region.  **Tanzania**: Geita district in Mwanza region, Biharamulo district in Kagera region, Bukombe, Shinyaga and Kahama district in Shinyaga region, the entire Mtwara region, Kilombero and Ulanga district in Morogoro region, Tunduru district in Ruvuma region, Liwale, Nachnigwea, Ruangwa and Lindi rural districts in Lindi region. Geita district, Mtwara and Lindi rural areas.  **DRC**: Equateur and Tshuapa district, Bongandanga and Bumba areas of Mongala district, Kungu and Budjala areas in Sud-Ubangi district all in the Equateur region. Mai-Ndombe district and Popukabaka in the Bandundu region, Bas-Fleuve and Cataractes districts in Bas-Congo region, Madimba and Kimvula in Kinshasa city region. Orientale region, Tshopo district specifically with the exception of Banalia and Bafwasende areas, Maniema district, Walikale and Lubero in Nord-Kivu district, Shabunda and Mwenga in Sud-Kivu district in the Kivu region. Sankuru district in the Kasai-Oriental region, Kasai, Kananga and Lulua in the Kasai-Occidental region, Kapanga area, Lualaba district in the Katanga region.  **Burundi**: Itaba, Makebuko, Buraza, Bukirasazi,  Giheta, Bugendana, Gitega and Mutaho districts in Gitega region.  **Uganda**: Bukolo district in Bugiri region, Kigulu and Bugweri in Iganga region.  **Zambia**: Kasama district in Northern region, Samfya district in Luapula region, Masaiti and Lufwanyama districts in the copperbelt region, Mufumbwe, Kabompo and Mwinilunga district in the Northwestern region  **Central African Republic**: Nola and Bambio districts in Sangha-Mbaere  **Mozambique**: Gorongosa and Gondola districts in Sofala region, all districts in Zambezia region with the exception of Morrumbala and Milange district in Zambezia region. The entire regions of Delgado and Nampula. Nipepe, Marrupa, Mecula, Mavago, Majume, Maua and Cuamba districts in the Nassa region  **Madagascar**: Atsinanana district in Toamasna region, Sava district in Antsirarana region | **Guinea**: Boke district in the Boke region  **Liberia**: GrandKru, Sinoe, Maryland, River Gee, Grand Gedeh, River Cess. GrandKru, Sinoe, Maryland, River Gee.  **Cote d’Ivoire**: Tabou district in the Bassandra region  **Benin**: Save district in Collines region  **Nigeria**: The whole of Bayelsa and Rivers States, Ayedaade, Irewole, Ayedire, Ife, Ede South, Egbedore, Ilesha East and Oriade areas of Osun State which make up about half of the LGAs in Osun State, The whole of Akwa Ibom State except Ikot Ekpene, Ika and Itu LGAs, Ikorodu, Epe, Lekki, Eti Osa, Lagos Island areas of Lagos State, The whole of Oyo State except Ibarapa, Saki, Orelope, Irepo and Ogbomoso North LGAs, the whole of Ogun state with the exception of Ipokia, Ifo, AdoOdo, Yewa and Abeokuta LGAs. The whole of Ondo State exept Akure, Owo, Ose and Akoko LGAs,Ovia, Ikpoba, Uhunmwonde, Orhionmwonde LGAs in Edo state, Kwande LGA in Benue State, Warri, Sapele, Ethiope, Okpe, Burutu, Bomadi, Patani, Ughelli South, Isoko South, Ukwuani, Ndokwa East, Ika South LGAs in Delta State, Ohaji and Ngor in Imo State, Ukwa, Ugwunagbo, Osisioma Ngwa and Oboma Ngwa LGAs in Abia State, Obanliku, Boki, Akamkpa, Odukpani, Akpabuyo, Calabar, Bakassi LGAs in Cross River State.  **Cameroon**: The entire Littoral, Sud-Ouest and Sud region, Mezam, Momo and Ngo Ketunja districts in the Nord-Ouest region, Menoua and Bamboutos in the Ouest region, Nyong et Kelle, Nyong at So'o, Nyong et Mfoumou, Mefou et akono, Mfoundi, Mefou at Afamba, Haut Nyong and Boumba et Ngoko district in the Est region**.**  **Equatorial Guinea:** The entire country from Litoral to Kie-Ntem, Wele-Nzas to Centro.  **Gabon**: The entire country except Lebounbi-Leyou, Mpassa and Plateaux districts in Haut- Ogooue region.  **Congo**: The entire country except Mindouli, Boko and Kinkala districts in the Pool region. Mbomo and Kelle districts in Cuvette-Ouest, Niari, Lekoumou and Kauilou regions.  **Angola**: The entire Cuaza Norte and Uige region, M'banza Congo and Cuimba in the Zaire region, Cacuzo, Malanje, Calandula, Massango and Luquembo districts in Malanje region,Nambuangongo, Dande, Icolo e Bengo districts in the Bengo region, Mussende in Cuanza Sul region, Xa Muteba in Lunda Norte region, Cameia, Moxico, Alto Zambeze and Lumbala-Nguimbo in the Moxico region.  **Mozambique**: The entire Cabo Delgado region except Balama and Muidumbe district, the entire Nampula region except Murrupula, Malema, Lalaua, Memo and Nacala Vulha district, Gile district in Zambezia region.  **DRC**: Equateur and Tshuapa district, Bongandanga area of Mongala district, Kungu and Budjala areas in Sud-Ubangi district all in the Equateur region, Boma, Lukaya, Bas-Fleuve and Cataractes districts in Bas-Congo region, Mai-Ndombe district in the Bandundu region. Kale, Lomela and Katako-Kombe areas in Sankuru district in the Kasai-Oriental region, Dekese and Luebo in Kasai, Kananga, Demba, Dimbelenge and Kazumba in the Lulua district, in the Kasai-Occidental region. Pangi, Kindu, Punia, Kibombo and Kasongo areas of Maniema district, Walikale, Rutshiru and Lubero in Nord-Kivu district, Shabunda, Kabare, Kaleha and Mwenga in Sud-Kivu district in the Kivu region. Kapanga area, Lualaba district in the Katanga region. Yohuna, Isangi, Opala and Ubundu areas of Kisangani district in the Orientale region. The following areas have high-high clusters of 0.001 significance. Tshela in Bas-Flueve district, Inongo in Mai-Ndombe, Monkoto and Bokungu in Tshuapa region, Kindu and Punia areas of Maniema district, Walikale and Rutshiru in Nord-Kivu district, Shabunda, Kabare, Kalehe and Mwenga in Sud-Kivu district in the Kivu region. |
| Significant   hotspots locations, p<0.001 | Cameroon, Equatorial Guinea, Gabon, Angola, Tanzania, Madagascar. | Liberia, Nigeria, Cameroon, Equatorial Guinea, Gabon, Congo. | **Cameroon:** Most of areas with high co-infections have high clusters of a high significance.  **Equatorial Guinea:** The entire country from Litoral to Kie-Ntem, Wele-Nzas to Centro Sur have high clusters of a high significance.  **Gabon**: Wouleu-Ntem, Estuaire, Ogooue et des Lacs district in Moyen-Ogooue, Mvoung district in Ogooue-Ivindo region, Sebe-Brikolo and lekoko district in Haut- Ogooue region.  **Angola**: All of the Zaire region with the exception of Soyo district. Maquela do Zombo, Damba, Buengas, Bembe, Quitexe and Puri districts in the Uige region and Bolongongo district in Cuaza Norte region have high clusters of a high significance  **Tanzania**: Geita district, Mtwara and Lindi rural areas.  **Madagascar:** Atsinanana district in Toamasna region, Sava district in Antsirarana region. | **Liberia**: GrandKru, Sinoe, Maryland, River Gee, Grand Gedeh, River Cess. GrandKru, Sinoe, Maryland, River Gee.  **Nigeria**: The whole of Bayelsa State, Ahoada, Abua, Degema Khana, Andoni, Obio Akpor, Ogu/Bolo in Rivers State,Warri, Sapele, Okpe, Burutu, Bomadi, Patani, Ughelli South, Ndokwa East LGAs in Delta State, Ondon West, Odigbo and Ilaje areas of Ondo State, Ijebu area of Ogun State, Epe in Lagos State, Oluyole, Egbeda and Ona Ara areas of Oyo State, Ayedaade, Irewole, Ayedire, Ife areas of Osun State.  **Cameroon**: The entire Littoral, Sud-Ouest and Sud region, Mezam, Momo and Ngo Ketunja districts in the Nord-Ouest region, Menoua and Bamboutos in the Ouest region, Nyong et Kelle, Nyong at So'o, Nyong et Mfoumou, Mefou et akono, Mfoundi, Mefou at Afamba, Haut Nyong and Boumba et Ngoko district in the Est region**.**  **Equatorial Guinea:** The entire country from Litoral to Kie-Ntem, Wele-Nzas to Centro.  **Gabon**: The entire country except Lebounbi-Leyou, Mpassa and Plateaux districts in Haut- Ogooue region.  **Congo**: The entire country except Mindouli, Boko and Kinkala districts in the Pool region. Mbomo and Kelle districts in Cuvette-Ouest, Niari, Lekoumou and Kauilou regions. |
| ***Plasmodium falciparum* and hookworm co-infection** | | | | |
|  | **Pre 2003 (ADM1=Countries)** | **2003-2018 (ADM1=Countries)** | **Pre 2003 (ADM2/ADM3)** | **2003-2018 (ADM2/ADM3)** |
| **High-high** prevalence of p. Falciparum and hookworm. | Benin, Cote d'Ivoire, Ghana, Guinea, Guinea Bissau, Liberia, Nigeria, Togo, Sierra Leone, Cameroon, Democratic Republic of the Congo, Equatorial Guinea, Gabon, Uganda, Tanzania, Angola, Mozambique, Madagascar, and Zambia. | Benin, Cote d'Ivoire, Nigeria, Togo, Ghana, Liberia, Guinea, Angola, Cameroon, DRC, Equatorial Guinea, Gabon, Uganda, Tanzania, Zambia, Mozambique. | **Nigeria**: Shendam in Plateau State, Kuje in Abuja, The whole of Abia, Akwa Ibom, Crossriver, Bayelsa, Rivers, Benue States. The whole of Ebonyi except Abakaliki, Ezza South and Ikwo LGAs, Ibi, Agatu, Wukari, Donga, Takum, Kurmi AND Ussa un Taraba State. Bassa, Omala, Ankpa in Kogi State. Nkanu East anad Isi Uzo LGAs in Enugu. Ovia South-West, Orhionmw and Ovia North LGA in Edo State. Ogun Water side in Ogun State, Odigbo, Okitipupa, Irele, Idanre in Ondo State. Barute LGA in Kware State. Kwande in Benue, Odukpani in cross Rivers State, Obio-Ngwa and Isiala Ngwa North LGA of Abia State. Warri North, Burutu,Bomadi, Patani, Ndokwa East in Delta State.  **Ghana**: Most of the districts East of the river volta including Bunkpurugu Yunyoo, Saboba Chereponi, Zabzugu Tatale,Nanumba South, Nkwanta, Kadjebi,Jasikan, Hohoe and Ho districts. Nkwanta, Kadjebi,Jasikan and Hohoe.  **Togo**: The entire country except Northwest area of Tone district and Lome district  **Benin**: The entire Donga, Collines, Zou and Kouffo regions,Toffo, Ze and Allade in Atlantique region, Bonou in Oueme region, All of Atakora except Kerou district, Mono region except Come district, and Borgou region except Bembereke, Nikki anad Kalale districts.  **Cote d’Ivoire**: Bondoukou in Zanzan region, all of Savanes except Northern area of Ferkessedougou,most parts of denguele region. All of Bafing and worodougou region, Dabakala and Katiola districts in Valee du Bandama region, Mbahiakro and southern parts of Dimbokro district in Nzi-Combe region, Lakota district in Sud-Bandama region, Soubre in the Bas-Sassandra, Daloa and Vavoua in Haut-Sassandra, Zuenoula district in Marahoue region.  **Cameroon**: All districts in the Sud Ouest region, Menchum, Boyo, Donga Mantung, Boyo, Momo and Mezam districts in Nord-Ouest region, Moungo, Nkam and Sanaga Maritime district in Littoral region, Ocean and Vallee du Ntem district in Sud region  **Liberia**: The whole of Liberia.  **Sierra Leone**: The whole country except Bonte and Pujehun district.  **Guinea Bissau**: western parts of Gabu district, Northern Bafata and southern Contuboel  **Guinea**: Boffa and Boke districts in Boke Region, Telimele, Forecariah and Dubreka district in Kindia region,Kankan and Kerouane didtrict in Kankan region, Kissidougou and Faranah districts in Faranah region and the entire Nzerekore region  **Equatorial Guinea**: Southern littoral, Centro Sur and Wele-Nzas region  **Gabon**: Komo and Noya districts in the Estuaire region, Haut-Como and Okano districts in the Wouleu-Item region  **DRC**: Kisangani,Tshopo, some parts of Ituri aad Haut-Uele district in Orientale region, parts of Mongala and Tshuapa district in Equateur region, All of Maniema, Kananga, Lulua district, some parts of Sud-Kivu, Nord-Kivu, Sankuru, Kasai, Lualaba and Tanganika district  **Uganda**: Iganga, Kamuli, Pallisa, Tororo, Western Mbale, Masaka, Buguri, Mayuge and Jinga regions. Masaka, Buguri and Iganga regions.  **Tanzania**: Bukombe and Kahama district in Shinyaga region, the entire Lindi and Mtwara region, the whole of Tabora region with the exception of Urban Tabora and Igunga district, Kibonda district in Kigoma region, Mpanda district in Rukwa region, Tunduru district in Ruvuma region, Kilombero and Ulanga district in Morogoro region  **Mozambique**: Most parts of Zambezia and Nassa regions, The entire regions of Delgado and Nampula and Maringue, Caia, Cheringoma, Gorongosa and Gondola districts in Sofala region  **Angola**: Lucano and Alto Zambeze districts in Moxico Region, Cacolo, Saurimo and Muconda districts in Lunda Sul region, and Alto Zambeze district.  **Zambia**: The entire Northern region, Kabompo, Mufumbwe, Kasempa, Solwezi and Mwinilunga district in North-Western region, Mpongwe, Masaiti, Luanshya and Lufwanyama districts in the copperbelt region, Kapiri Mposhi and Serenje, Mkushi district in Central region, Mpika district in Muchinga region,Petauke and Mambwe district in Eastern region. Most of the Northern region, Kabompo, Mufumbwe, Kasempa and Mwinilunga district in North-Western region | **Nigeria**: The whole of Akwa Ibom, Bayelsa, Rivers, Benue States (with the exception of Agatu LGA), The whole of Abia State (with the exception of Umuahia North and South, Isikwuato and Umuneochi LGA), Takum in Taraba State, Doma and Awe LGAs in Nasarawa State, Akampka, Calabar, Biase, Odukpani, Akpabuyo, Boki, Obudu, Obanliku LGAs in Cross River State, Ohaozara LGA in Ebonyi State, Ngor, Ohaji and Oguta LGAs in Imo State. Warri-North,Warri South-West,Ethiope-West, Ethiope East, Sapele, Isoko South,Ughelii South, Burutu,Bomadi, Patani, Ndokwa East in Delta State. Ovia South-West, Orhionmwon, Ikpoba and Ovia North LGA in Edo State, Ogun Water side and Ijebu LGAs in Ogun State, Odigbo, Okitipupa, Irele, Ilaje and EseOdo in Ondo State, Olamabor and Ankpa in Kogi State, Egbeda in Oyo State, Irewole and Ayedaade in Osun State. All but three LGAs in Yenegoa and Kolokuma/Opokuma in Bayelsa State. Degema, Khana and Abua in Rivers State and Ughelli South in Delta State.  **Benin**: The entire Donga region, Savalou, Bante, Ouesse and Glazoue districts in Collines, Djidja district in Zou region, Aplahoue, Djakotomey, Dogbo and Klouekanme districts in Kouffo region,Tchaourou district in Borgou region, Athieme district in Mono region, Kouande and Boukounmbe districts in Atakora region.  **Togo:** The entire country except Northwest area of Tone district and Lome district.  **Cote d’Ivoire**: Touba, Biankouma and Man districts in the Dix-Huit Montagnes region.  **Ghana**: East of the river volta including Zabzugu Tatale,Nanumba South,Nkwanta, Kadjebi,Jasikan, Hohoe and Ho districts.  **Liberia**: GrandKru, Sinoe, River Gee, River Cess, Maryland, Nimba, Lofa, Gbapolu, Grand Cape Mount, Bomi, Montserrado, Margibi region. GrandKru, Sinoe, River Gee and Maryland. Lofa, Gbapolu, Grand Cape Mount, Bomi, Montserrado, Margibi, River Cess and Nimba regions.  **Guinea**: Fria and Boke districts in Boke Region.  **Cameroon**: Sanaga Maritime district in Littoral region, Ndian and Manyu districts in the Sud Ouest region. Momo district in Nord-Ouest region. Vallee du Ntem in the Sud region.  **Equatorial Guinea**: Southern littoral, Centro Sur and Wele-Nzas region  **Gabon**: Noya district in the Estuaire region, Haut-Como and Okano districts in the Wouleu-Ntem, Lope in Ogooue-Ivindo region, Lolo-Bouenguidi and Lombo-Bouenguidi in Ogooue-Lolo region.  **Uganda**: Kamuli, Iganga, Tororo, Mbale, Sironko, Jinja, Mayuge, Masaka, Bugiri, Palissa, Kumi, Kapchorwa regions.  **Tanzania**: Urambo district in Tabora region  **Zambia**: Chilubi, Luwingu, Mporokoso, Kaputa districts in Northern region.  **Mozambique**: The entire regions of Delgado and Nampula, Muembe, Maua, Marrupa, Majune and Npepe districts in Nassa region, Mopela, Mocuba, Pebane, Ile, Gile, Gurue,n Alto Molocue, Namacurra, Maganja da costa, Nicoadala districts in Zambezia region.  **DRC**: Kananga, Lulua district, Luebo area of Kasai district, Kamiji, Tshilenge, Mwene-Ditu areas of Kabinda district, Kapanga area of Lualaba, Lubao area of Moba and Kongolo area of Tanganika district, Kindu, Kibombo, Pangi, Kasongo and Punia areas of Maniema district, Shabunda, Mwenga, Kabare, Kalehe areas in Sud-Kivu district, Walikale district in Nord-Kivu district, Lomela area in Sankuru district, Opala, Yohuma, Isangi areas in the Tshopo district, Bongandanga area of the Mongala district, Djolu, Monkoto, Ikela and Bokungu areas of the Tshuapa district.  **Angola**: All districts except leua in Moxico Region, Cacolo and Dala districts in Lunda Sul region, Lucapa and Cambulo district in Lunda Norte region, Mavinga, Nancova, Menogue, Cuito Cuanavale districts in the Cuando Cubango region. |
| Significant   hotspots locations, p<0.001 | Ghana, Liberia, Nigeria, Sierra Leone, Angola, Uganda, Zambia. | Guinea and Nigeria. | **Nigeria**: Kwande in Benue, Odukpani in cross Rivers State, Obio-Ngwa and Isiala Ngwa North LGA of Abia State. Warri North, Burutu,Bomadi, Patani, Ndokwa East in Delta State  **Ghana**: Nkwanta, Kadjebi,Jasikan and Hohoe,  **Liberia:** GrandKru, Sinoe, River Gee and Grand Gedeh, Gbapolu and Bong regions.  **Sierra Leone**: Moyambe district.  **Uganda**: Iganga, Kamuli, Pallisa, Tororo, Western Mbale, Masaka, Buguri, Mayuge and Jinga regions. Masaka, Buguri and Iganga regions.  **Angola**: Alto Zambeze district  **Zambia**: Most of the Northern region, Kabompo, Mufumbwe, Kasempa and Mwinilunga district in North-Western region | **Nigeria**: All but three LGAs in Yenegoa and Kolokuma/Opokuma in Bayelsa State. Degema, Khana and Abua in Rivers State and Ughelli South in Delta State.  **Guinea**: Boke |
| ***Plasmodium falciparum* and A*scaris lumbricoides* co-infection** | | | | |
|  | **Pre 2003 (ADM1=Countries)** | **2003-2018 (ADM1=Countries)** | **Pre 2003 (ADM2/ADM3)** | **2003-2018 (ADM2/ADM3)** |
| **High-high** prevalence of p. Falciparum and Ascaris lumbricoides | Liberia, Nigeria, Cameroon, Equatorial Guinea, Gabon, Congo, Angola, Burundi, Democratic Republic of the Congo, Kenya, Mozambique, Madagascar. | Liberia, Nigeria, Cameroon, DRC, Equatorial Guinea, Gabon, Congo, Angola, Burundi, Kenya, Mozambique. | **Liberia**: The entire country except Lofa, Gbapolu and Nimba regions. River Gee, Sinoe River Cess and Grand Kru regions.  **Nigeria**: The whole of Bayelsa, Ekiti and Osun states, the whole of Ondo State except Irele LGA, most parts of Ogun, Oyo and Kwara States. Akampka, Akpabuyo, odukpani and Calabar areas of Cross River state, Ndokwa and Isoko areas of Delta state, Eleme, Khana, Gokana, Tai, Opobo, Nkor, Bonny, Okrika, Degema, Ahoada LGAs of Rivers State, Owan West, Ovia North and Ovia South West in Edo State.  **Cameroon**: the entire Littoral, Sud-Ouest and Sud region, Menchum, Momo, Donga Matung and Ngo Ketung distrist in Nord-Ouest region. The entire littoral region and Ndian, Meme and Fako districts in Sud-Ouest region.  **Equatorial Guinea**: The entire country from Litoral to Kie-Ntem, Wele-Nzas to Centro Sur.  **Gabon**: Wouleu-Ntem, Estuaire, Moyen-Ogooue, Mvoung district in Ogooue-Ivindo region, Basse Banio in Nyanga region,Sebe-Brikolo and lekoko district in Haut- Ogooue region.  **Congo**: Likoula, Sangha, Cuvette, Cuvette Ouest, Plateaux, lekoumou regions, Mayama, Ngabe and Ngamaba districts in the Pool region, Divene and Kibangou district i the Nari region, Madingo Kayes, Mvouti and Kakamoeka districts in the Koillou region  **Angola**: The entire Cuaza Norte and Uige region, Cacuzo, Cuaba, Calandula, Massango districts in Malanje region, Ambriz, Nambuangongo, Dande, Icolo e Bengo districts in the Bengo region. All of the Zaire region with the exception of Soyo district. The entire Cuaza Norte and Uige region and Cuimba, Mbanza'Congo and Nambuangongo.  **Kenya**: Marani area of Kisii district ,Ekerenyo of Nyamira district  **Burundi**: Shombo, Gihagazi, Nyabikere, Mutumba districts in Karuzi region, Butezi and Butaganzwa in Ruyigi region, Itaba, Makebuko, Buraza, Bukirasazi, Giheta, Bugendana, Gitega and Mutaho districts in Gitega region. Giheta,Gitega, Bugendana and Mutaho districts  **DRC**: All of Mai-Ndombe, Lukaya, Kinshasa, Tshaupa, Cataractes, Bas-Flueve, Boma, Matadi and Kasai districts.  Lubumbashi and Kipushi in Haut-Shaba district. Mbandaka,Bandundu districts, Businga in Nord-Ubangi districts, the bulk of central parts of Kivu which includes Kindu, Pangi and Punia areas of Maniema district, Shabunda, Mwenga, Kabare, Kalehe areas in Sud-Kivu district, Walikale and rutshiru, district in Nord-Kivu district, Lomela , Katako-Kombe, Lomela and Kole in Sankuru district, All of Equateur region except Nord Ubangi, Northern parts of Sud Ubangi and Northern parts of Mongala, Ubundu in Shopo district.  **Mozambique**: Magude and Manhica districts in Maputo,Chokwe, Bilene, Xai-xai and Mandlakazi in the Gaza region, Inharrime, Homoine and Jangamo in Inhambane  **Madagascar**: Atsinanana district in Toamasna region, Sava district in Antsirarana region. | **Liberia**: The entire country except Lofa, Gbapolu, Margibi, River Cess, Grand Bassa,Bomi, Grand Cape Mount and Nimba regions.  **Nigeria**: The whole of Bayelsa, Half the LGAs in Akwa Ibom State, Akampka, Etung, Akpabuyo, odukpani and Calabar areas of Cross River state. The whole of Osun state with the exception of Irepodun LGA, Oyo State with the exception of Irepo, Saki East and Saki West, Ogun State with the exception of AdoOdo/Ota, The whole of Ondo state with the exception of Akoko North-West, The whole of Ekiti state with the exception of Ekiti East LGA, The whole of Kwara state with the exception of Isin, Offa, Edu and Pategi LGAs. Ndokwa, Ughelli, Warri, Sapele, Ethiope, Isokom Bomadi and Burutu areas of Delta state, The whole of Rivers state with the exception of Ikwerre, Etche, ukwa West, Port Harcourt, Ommuma and Oyigbo. The following LGAs in Lagos State, Epe, Ibeju/Lekki, Eti Osa, Ikorodu, Lagos Island, Lagos Mainland, Oshodi/Isolo, Mushin, Shomolu, Ikeja, Kosofe and Surulere. The following LGAs in Edo State, Owan West, Ovia North and Ovia South West, Uhunmwode, Ikpoba-Okha, Oredo, Egor.  **Cameroon**: Nyong et Kelle and Nyong et So'o in the Centre region of Cameroon, The entire Littoral region except for the Ndom and Ngambe area, the entire Sud-Ouest region with the exception of the Akwaya area, the entire Sud region with the exception of the Northern parts of Dja et Lobo.  **Equatorial Guinea**: The entire country  **Gabon**: The entire country except Leboumbi-Leyou, Mpassa and Plateaux districts in Haut-Ogooue region  **Congo**: the entire country except Mindouli, Boko and Kinkala districts in the Pool region  **Angola**: The entire Cuaza Norte and Uige region.Cacuzo, Calandula, Massango districts in Malanje region, Nambuangongo, Dande, Icolo e Bengo districts in the Bengo region. All of the Zaire region with the exception of Soyo district.  **Burundi**: Buezi and Butaganzwa districts.  **Kenya**: Marani area of Kisii district.  **Mozambique**: Homoine district in Inhambane region.  **DRC**: All of Mai-Ndombe, Lukaya, Cataractes, Bas-Flueve, Boma, Matadi and Kasai districts. Kipushi in Haut-Shaba district. Kindu, Pangi and Punia areas of Maniema district, Shabunda, Mwenga, Kabare, Kalehe areas in Sud-Kivu district, Walikale and rutshiru, district in Nord-Kivu district,Lomela , Katako-Kombe and Kole in Sankuru district. All of Equateur region except Nord Ubangi, Northern parts of Sud Ubangi and Northern parts of Mongala, Ubundu in Shopo district. |
| Significant   hotspots locations, p<0.001 | Liberia, Cameroon, Equatorial Guinea, Angola, Burundi, Madagascar. | Cameroon, Equatorial Guinea, Angola. **3 countries** | **Liberia:** The entire country except Lofa, Gbapolu and Nimba regions. River Gee, Sinoe River Cess and Grand Kru regions  **Cameroon**: The entire littoral region and Ndian, Meme and Fako districts in Sud-Ouest region  **Equatorial Guinea:** The entire country from Litoral to Kie-Ntem, Wele-Nzas to Centro Sur  **Angola: T**he entire Cuaza Norte and Uige region and Cuimba, Mbanza'Congo and Nambuangongo  **Burundi**: Giheta,Gitega, Bugendana and Mutaho districts  **Madagascar**: Atsinanana district in Toamasna region, Sava district in Antsirarana region. | **Cameroon**: Ndian, Meme, Nkam and Sanaga Maritime districts  **Equatorial Guinea**: Wele-Nzas to Centro Sur  Angola: Half of the districts in Cuaza Norte. The entire Uige region with the exception of Songo, Ambuila and Quimbele district. Also, Dande and Nambuangongo in the Bengo. |
| ***Plasmodium falciparum* and *Trichuris trichiura* co-infection** | | | | |
|  | **Pre 2003 (ADM1=Countries)** | **2003-2018 (ADM1=Countries)** | **Pre 2003 (ADM2/ADM3)** | **2003-2018 (ADM2/ADM3)** |
| **High-high** prevalence of p. Falciparum and Trichuris Trichiuria. | Liberia, Cameroon, Equatorial Guinea, Gabon, DRC, Congo, Angola, Burundi, Kenya, Madagascar. | Cameroon, Equatorial Guinea, Gabon, Congo, DRC. | **Liberia**: River Gee, Maryland and Grand Kru regions.  **Cameroon**: All of Centre region except Mbam et Kim and Haute Sanaga districts, the entire Littoral, Sud-Ouest and Sud region, Haut Nkam and Nde districts in the Ouest region and Haut-Nyong district in Est region.  **Equatorial Guinea**: The entire country from Litoral to Kie-Ntem, Wele-Nzas to Centro Sur.  **Gabon**: Wouleu-Ntem, Estuaire, Ogooue et des Lacs district in Moyen-Ogooue, Mvoung district in Ogooue-Ivindo region, Sebe-Brikolo and lekoko district in Haut- Ogooue region.  **DRC**: Ubundu and Bafwasende in the Tshopo district in Orientale region, Lubero, Rutshuru and Walikali areas in North Kivu district,Punia in Maniema district,Shabunda and Kabare in Sud-Kivu district all within the Kivu region, Mankoto, Bokungu, Boende and Befale areas in the Tshuapa district, the entire Equateur district except Basankusu, Kole and Lomela in Sankuru district, Dekeseand Mweka areas of Kasai district, the entire Mai-Ndombe district except areas in Mushie, the entire Bas-Fleuve and Cataractes districts save for some areas in Mbanza-Ngungu  **Congo**: The entire Sangha, Cuvette, Cuvette Ouest, Plateaux regions, Epena nad Impfondo districts in Likouala region, Mayama, Ngabe and Ngamaba districts in the pool region, Madingo-Kayes, Kakamoeka and Mvouti districts in the Kouilou region, Kibangou and Divene district in the Niari region.  **Angola**: Dande and Nambuangongo districts in Bengo region, Noqui distrcit in Zaire region, Dembos district in Cuaza Norte region, Ambuilla and Quitexe district in Uige region  **Burundi**: Bugendana and Mutaho districts in Gitega region.  **Kenya**: Kwale district in the Coast regionof Kenya  **Madagascar**: Atsinanana district in Toamasna region, Sava district in Antsirarana region. | **Cameroon**: All of Centre region except Mbam et Kim and Haute Sanaga districts. The entire Littoral and Sud region, Haut-Nyong district in Est region, Nde district in the Ouest region, the entire Sud-Ouest with the exception of Eyumodjock and Akwaya districts.  **Equatorial Guinea**: The entire country from Litoral to Kie-Ntem, Wele-Nzas to Centro Sur.  **Gabon**: The entire country except Lebounbi-Leyou, Mpassa and Plateaux districts in Haut- Ogooue region.  **Congo**: The entire country except all of the Pool region and Dongou district in Likouala region  **DRC:** Boma, Bas-Fleuve and parts of Cataractes districts, Dekese in Kasai district, Kole in Sankuru district, Ubundu in Tshopo district, Walikale and Rutshiru districts in Nord-Kivu district, North-Eastern parts of Shabunda in Maniema district, Oshwe, Inongo and Kiri areas in Mai-Ndombe district, Lukolela, Bikoro, Bolomba, Ingende in Equateur, Buende and Monkoto in Tshuapa district. |
| Significant   hotspots locations, p<0.001 | Cameroon, Equatorial Guinea, Gabon, Madagascar. | Cameroon, Equatorial Guinea, Gabon. | **Cameroon**: All of Centre region except Mbam et Kim and Haute Sanaga districts, the entire Littoral, Sud-Ouest and Sud region, Haut Nkam and Nde districts in the Ouest region and Haut-Nyong district in Est region.  **Equatorial Guinea**: The entire country from Litoral to Kie-Ntem, Wele-Nzas to Centro Sur.  **Gabon**: Wouleu-Ntem, Estuaire, Ogooue et des Lacs district in Moyen-Ogooue, Mvoung district in Ogooue-Ivindo region, Sebe-Brikolo and lekoko district in Haut- Ogooue region.  **Madagascar**: Atsinanana district in Toamasna region, Sava district in Antsirarana region. | **Cameroon**: Half the area in the Sud region, major parts of Littoral region and the Meme district in Sud-Ouest.  **Equatorial Guinea**: The entire country from Litoral to Kie-Ntem, Wele-Nzas to Centro Sur.  **Gabon**: The entire country except Lebounbi-Leyou, Mpassa and Plateaux districts in Haut- Ogooue region.  **Congo**: The entire country except all of the Pool region and Dongou district in Likouala region |

**Note:** ADM1 = first administrative level, ADM2 = second administrative level, ADM3 = third administrative level, DRC = Democratic Republic of the Congo
