# Supplementary material for: Prevalence and distribution pattern of malaria and soil-transmitted helminth co-endemicity in sub-Saharan Africa, 2000–2018: A geospatial analysis
Source: PLoS Negl Trop Dis. 2022 Sep 30;16(9):e0010321. doi: 10.1371/journal.pntd.0010321 (PMC9555675; doi:10.1371/journal.pntd.0010321)
Supplement: S2 Table — (DOCX) [file pntd.0010321.s006.docx]

**S2 Table**: Summary table of STH prevalence estimates by country and frequency of prevalence surveys per country, sub-Saharan Africa, 2000-2018

| **Country** | **Year** | **Number of prevalence surveys** |
| --- | --- | --- |
| Angola | 2010 | 39 |
| Benin | 2000 | 1 |
|  | 2005 | 2 |
|  | 2009 | 1 |
|  | 2012 | 16 |
|  | 2013 | 17 |
|  | 2014 | 150 |
|  | 2015 | 195 |
|  | 2017 | 82 |
| Burkina Faso | 2004 | 87 |
|  | 2013 | 7 |
| Burundi | 2007 | 21 |
|  | 2014 | 110 |
| Botswana | 2015 | 128 |
| Cameroon | 2000 | 1 |
|  | 2001 | 1 |
|  | 2002 | 2 |
|  | 2010 | 2 |
|  | 2011 | 2 |
|  | 2012 | 137 |
| Cape Verde | 2005 | 6 |
|  | 2012 | 7 |
| Chad | 2000 | 19 |
|  | 2008 | 2 |
|  | 2015 | 338 |
| Cote d’Ivoire | 2000 | 1 |
|  | 2001 | 1 |
|  | 2002 | 1 |
|  | 2006 | 4 |
|  | 2007 | 1 |
|  | 2008 | 2 |
|  | 2009 | 1 |
|  | 2010 | 10 |
|  | 2011 | 3 |
|  | 2012 | 1 |
|  | 2013 | 1 |
| DR Congo | 2009 | 1 |
|  | 2012 | 708 |
|  | 2013 | 818 |
|  | 2014 | 233 |
|  | 2015 | 108 |
| Ethiopia | 2000 | 1 |
|  | 2001 | 1 |
|  | 2003 | 5 |
|  | 2005 | 165 |
|  | 2008 | 4 |
|  | 2009 | 108 |
|  | 2010 | 9 |
|  | 2011 | 3 |
|  | 2012 | 7 |
|  | 2013 | 10 |
|  | 2014 | 2 |
|  | 2015 | 3 |
| Eritrea | 2002 | 40 |
|  | 2014 | 170 |
|  | 2015 | 163 |
| Gabon | 2014 | 61 |
|  | 2015 | 182 |
| Ghana | 2007 | 1 |
|  | 2008 | 74 |
|  | 2010 | 13 |
|  | 2012 | 1 |
| Guinea | 2013 | 23 |
| Guinea-Bissau | 2001 | 1 |
|  | 2004 | 3 |
| Kenya | 2000 | 8 |
|  | 2001 | 31 |
|  | 2002 | 18 |
|  | 2004 | 143 |
|  | 2005 | 62 |
|  | 2006 | 50 |
|  | 2007 | 73 |
|  | 2008 | 158 |
|  | 2009 | 167 |
|  | 2010 | 12 |
|  | 2011 | 9 |
|  | 2012 | 348 |
|  | 2013 | 10 |
| Mali | 2004 | 1 |
|  | 2005 | 1 |
|  | 2007 | 1 |
|  | 2014 | 14 |
|  | 2015 | 8 |
|  | 2016 | 8 |
| Malawi | 2002 | 29 |
|  | 2004 | 1 |
|  | 2012 | 251 |
|  | 2013 | 74 |
| Madagascar | 2001 | 1 |
|  | 2008 | 2 |
|  | 2010 | 1 |
|  | 2011 | 2 |
|  | 2012 | 4 |
|  | 2013 | 5 |
|  | 2014 | 19 |
|  | 2015 | 248 |
| Mauritania | 2015 | 123 |
| Mauritius | 2015 | 47 |
| Mozambique | 2005 | 3 |
|  | 2007 | 118 |
|  | 2009 | 1 |
| Niger | 2004 | 141 |
|  | 2006 | 70 |
| Nigeria | 2000 | 1 |
|  | 2001 | 10 |
|  | 2002 | 4 |
|  | 2003 | 22 |
|  | 2004 | 12 |
|  | 2005 | 12 |
|  | 2006 | 15 |
|  | 2007 | 1 |
|  | 2008 | 6 |
|  | 2009 | 87 |
|  | 2010 | 145 |
|  | 2011 | 759 |
|  | 2012 | 117 |
|  | 2013 | 878 |
|  | 2014 | 702 |
| Rwanda | 2007 | 48 |
|  | 2008 | 209 |
|  | 2010 | 1 |
|  | 2014 | 186 |
| Sao Tome & Principe | 2009 | 2 |
|  | 2014 | 47 |
| Senegal | 2008 | 1 |
|  | 2009 | 4 |
|  | 2010 | 1 |
|  | 2013 | 105 |
| Sierra Leone | 2008 | 139 |
| South Africa | 2000 | 2 |
|  | 2001 | 4 |
|  | 2003 | 1 |
| Sudan (southern) | 2008 | 1 |
|  | 2009 | 75 |
|  | 2010 | 134 |
|  | 2016 | 119 |
| Swaziland | 2015 | 275 |
| Tanzania (Mainland) | 2000 | 2 |
|  | 2002 | 3 |
|  | 2004 | 141 |
|  | 2008 | 9 |
|  | 2009 | 1 |
|  | 2011 | 69 |
|  | 2012 | 4 |
|  | 2018 | 338 |
| Tanzania (Zanzibar) | 2000 | 5 |
|  | 2006 | 4 |
|  | 2007 | 10 |
|  | 2009 | 2 |
|  | 2011 | 37 |
|  | 2012 | 2 |
|  | 2013 | 2 |
| The Gambia | 2005 | 20 |
|  | 2010 | 28 |
|  | 2015 | 382 |
| Togo | 2009 | 1090 |
|  | 2015 | 1110 |
| Uganda | 2002 | 30 |
|  | 2004 | 24 |
|  | 2003 | 36 |
|  | 2005 | 48 |
|  | 2006 | 58 |
|  | 2007 | 13 |
|  | 2008 | 187 |
|  | 2009 | 279 |
|  | 2010 | 277 |
|  | 2011 | 382 |
|  | 2012 | 37 |
|  | 2013 | 82 |
| Zambia | 2002 | 31 |
|  | 2005 | 57 |
|  | 2007 | 17 |
|  | 2013 | 1 |
| Zimbabwe | 2004 | 4 |
|  | 2010 | 102 |
