# Supplementary material for: Prevalence and distribution pattern of malaria and soil-transmitted helminth co-endemicity in sub-Saharan Africa, 2000–2018: A geospatial analysis
Source: PLoS Negl Trop Dis. 2022 Sep 30;16(9):e0010321. doi: 10.1371/journal.pntd.0010321 (PMC9555675; doi:10.1371/journal.pntd.0010321)
Supplement: S3 Table — (DOCX) [file pntd.0010321.s007.docx]

**S3 Table:** Summary table of *P.falciparum* prevalence estimates by country and frequency of prevalence surveys per country, sub-Saharan Africa, 2000-2018

| **Country** | **Year** | **Number of prevalence survey** |
| --- | --- | --- |
| Angola | 2005 | 1 |
|  | 2006 | 1 |
| Burundi | 2002 | 12 |
|  | 2003 | 80 |
|  | 2004 | 88 |
|  | 2005 | 93 |
|  | 2006 | 51 |
| Benin | 2003 | 7 |
|  | 2004 | 2 |
|  | 2006 | 1 |
|  | 2007 | 7 |
|  | 2008 | 3 |
| Burkina Faso | 2000 | 20 |
|  | 2001 | 2 |
|  | 2002 | 5 |
|  | 2003 | 17 |
|  | 2004 | 20 |
|  | 2005 | 1 |
|  | 2007 | 7 |
|  | 2008 | 11 |
|  | 2009 | 4 |
|  | 2010 | 6 |
|  | 2011 | 16 |
|  | 2012 | 2 |
| Cote d’Ivoire | 2000 | 1 |
|  | 2001 | 1 |
|  | 2002 | 5 |
|  | 2003 | 4 |
|  | 2005 | 12 |
|  | 2007 | 12 |
|  | 2010 | 5 |
|  | 2011 | 3 |
|  | 2012 | 4 |
|  | 2014 | 9 |
|  | 2015 | 8 |
| Cameroon | 2000 | 1 |
|  | 2001 | 4 |
|  | 2002 | 3 |
|  | 2003 | 10 |
|  | 2004 | 4 |
|  | 2005 | 1 |
|  | 2006 | 7 |
|  | 2008 | 7 |
|  | 2011 | 5 |
|  | 2013 | 7 |
| DR Congo | 2000 | 5 |
|  | 2002 | 3 |
|  | 2009 | 8 |
|  | 2011 | 24 |
|  | 2012 | 2 |
|  | 2013 | 2 |
|  | 2014 | 1 |
| Congo | 2010 | 2 |
| Comoros | 2006 | 12 |
| Cape Verde | 2002 | 1 |
|  | 2003 | 4 |
| Eritrea | 2002 | 3 |
| Ethiopia | 2001 | 1 |
|  | 2003 | 4 |
|  | 2004 | 14 |
|  | 2006 | 4 |
|  | 2008 | 1 |
|  | 2009 | 235 |
|  | 2011 | 1 |
|  | 2012 | 18 |
|  | 2013 | 1 |
| Gabon | 2000 | 1 |
|  | 2013 | 4 |
|  | 2014 | 2 |
| Ghana | 2000 | 1 |
|  | 2002 | 55 |
|  | 2003 | 1 |
|  | 2004 | 2 |
|  | 2005 | 2 |
|  | 2006 | 1 |
|  | 2007 | 1 |
|  | 2010 | 3 |
|  | 2011 | 1 |
| Gambia | 2001 | 16 |
|  | 2002 | 1 |
|  | 2004 | 1 |
|  | 2006 | 37 |
|  | 2007 | 38 |
|  | 2008 | 11 |
| Guinea-Bissau | 2003 | 2 |
|  | 2008 | 4 |
| Equatorial Guinea | 2004 | 2 |
|  | 2014 | 24 |
| Kenya | 2000 | 44 |
|  | 2001 | 33 |
|  | 2002 | 41 |
|  | 2003 | 132 |
|  | 2004 | 37 |
|  | 2005 | 62 |
|  | 2006 | 81 |
|  | 2007 | 37 |
|  | 2008 | 94 |
|  | 2009 | 390 |
|  | 2010 | 255 |
|  | 2011 | 53 |
|  | 2012 | 1 |
|  | 2013 | 6 |
| Madagascar | 2001 | 1 |
|  | 2002 | 3 |
|  | 2003 | 8 |
|  | 2004 | 3 |
|  | 2005 | 2 |
|  | 2012 | 133 |
|  | 2013 | 16 |
| Mali | 2002 | 1 |
|  | 2004 | 8 |
|  | 2006 | 6 |
|  | 2007 | 1 |
|  | 2008 | 2 |
|  | 2010 | 2 |
|  | 2011 | 2 |
| Mozambique | 2001 | 1 |
|  | 2002 | 64 |
|  | 2003 | 65 |
|  | 2005 | 1 |
|  | 2007 | 346 |
|  | 2008 | 2 |
|  | 2009 | 2 |
|  | 2010 | 2 |
|  | 2011 | 2 |
| Mauritania | 2004 | 1 |
|  | 2005 | 1 |
|  | 2006 | 1 |
|  | 2011 | 11 |
|  | 2012 | 9 |
|  | 2013 | 9 |
| Malawi | 2002 | 2 |
|  | 2003 | 10 |
|  | 2005 | 14 |
|  | 2006 | 190 |
|  | 2009 | 14 |
|  | 2010 | 14 |
|  | 2011 | 50 |
|  | 2013 | 3 |
| Niger | 2004 | 2 |
|  | 2007 | 1 |
|  | 2008 | 1 |
|  | 2009 | 1 |
| Nigeria | 2000 | 1 |
|  | 2001 | 2 |
|  | 2002 | 5 |
|  | 2003 | 1 |
|  | 2004 | 1 |
|  | 2005 | 9 |
|  | 2006 | 2 |
|  | 2007 | 5 |
|  | 2008 | 3 |
|  | 2009 | 1 |
|  | 2011 | 1 |
|  | 2012 | 1 |
|  | 2013 | 5 |
| Rwanda | 2011 | 2 |
| Sudan | 2000 | 3 |
|  | 2001 | 2 |
|  | 2002 | 2 |
|  | 2004 | 2 |
|  | 2005 | 2 |
|  | 2006 | 1 |
|  | 2008 | 2 |
| Senegal | 2001 | 2 |
|  | 2002 | 11 |
|  | 2003 | 10 |
|  | 2004 | 4 |
|  | 2005 | 2 |
|  | 2007 | 2 |
|  | 2008 | 40 |
|  | 2009 | 15 |
|  | 2010 | 5 |
| Sierra Leone | 2003 | 2 |
| Somalia | 2002 | 2 |
|  | 2005 | 277 |
|  | 2007 | 443 |
|  | 2008 | 523 |
|  | 2009 | 346 |
| South Sudan | 2006 | 2 |
|  | 2012 | 1 |
| Sao Tome & Principe | 2004 | 2 |
| Chad | 2004 | 1 |
| Tanzania | 2000 | 54 |
|  | 2001 | 115 |
|  | 2002 | 73 |
|  | 2003 | 13 |
|  | 2004 | 279 |
|  | 2005 | 103 |
|  | 2006 | 265 |
|  | 2007 | 30 |
|  | 2008 | 156 |
|  | 2012 | 6 |
| Uganda | 2001 | 1 |
|  | 2002 | 4 |
|  | 2003 | 4 |
|  | 2004 | 1 |
|  | 2005 | 2 |
|  | 2006 | 32 |
|  | 2007 | 1 |
|  | 2008 | 4 |
|  | 2009 | 23 |
|  | 2010 | 75 |
|  | 2011 | 4 |
|  | 2015 | 5 |
| South Africa | 2000 | 3 |
| Zambia | 2003 | 9 |
|  | 2004 | 6 |
|  | 2005 | 8 |
|  | 2007 | 1 |
|  | 2008 | 53 |
|  | 2009 | 28 |
|  | 2010 | 17 |
|  | 2014 | 60 |
|  | 2015 | 60 |
|  | 2016 | 60 |
| Zimbabwe | 2002 | 2 |
|  | 2004 | 5 |
|  | 2005 | 1 |
|  | 2013 | 2 |
