# Supplementary material for: Prevalence and distribution pattern of malaria and soil-transmitted helminth co-endemicity in sub-Saharan Africa, 2000–2018: A geospatial analysis
Source: PLoS Negl Trop Dis. 2022 Sep 30;16(9):e0010321. doi: 10.1371/journal.pntd.0010321 (PMC9555675; doi:10.1371/journal.pntd.0010321)
Supplement: Attachment — Submitted filename: RESPONSE SHEET_PNTD-D-22-00324_final.doc [file pntd.0010321.s008.doc]

14 July 2022

Dear Editor,

Revision of manuscript PNTD-D-22-00324: Prevalence and distribution pattern of malaria and soil-transmitted helminth co-infection in sub-Saharan Africa, 2000-2018: a geospatial analysis.

Thank you for sharing with us the reviewers’ comments on the above named manuscript. We are pleased that the reviewers feel the manuscript is an interesting paper on an important topic, and we have carefully taken the editorial and reviewers’ comments and suggestions into account in the revision of the manuscript. We describe below how we have responded to specific comments.

We have addressed the concerns from the reviewers and editors, and hope this paper is now acceptable for publication in your journal.

Yours sincerely,

Muhammed Afolabi

On behalf of all co-authors

**Reviewers' comments:**

Reviewer #1: METHODS

1. Is the MAP data only individuals aged 5-14 years? Clarify. More information is needed on the prevalence estimates for MAP; provide key information on demographics, source of prevalence surveys, and concentration of data by country.

*Response: The Malaria Atlas Project (MAP) data are not for individuals aged 5-14 years only. We have added more information on the prevalence estimates for MAP in lines 173-190 as follows: To address the challenges of repeated prevalence estimation, the MAP initiative was established in 2006 to project the expected prevalence and endemicity of malaria in all locations around the world, by modelling the available prevalence data. The intensity of malaria transmission or endemicity was described based on the prevalence of peripheral malaria parasitaemia in children aged 2 to 10 years (PfPR_2–10_). This is because the index has been found to closely correlate with the entomological inoculation rate (EIR) which refers to the number of infectious bites per person per day. Due to the ability of PfPR_2–10_ to mirror the EIR, the MAP generated models to predict the PfPR_2–10_ for a given set of environmental conditions. In high-endemicity areas, parasite rate (PR) samples are often restricted to children aged 2-10 years, but in areas of low endemicity, surveys are usually extended to include all age groups.*

*MAP collated survey data that are typically clustered at village level and record data on parasite positivity rates. MAPs used data points generated from malaria surveys for its estimation by combining these data points with GIS data. MAP was updated in 2010 to include new malariometric data extending to 13,449 administrative units in 43 endemic countries and 22,212 Plasmodium falciparum parasite rate (PfPR) surveys were used to define spatial limits of malaria transmission.*

*To generate continuous global maps, MAP matched spatially referenced data, e.g., altitude, temperature, rainfall, and vegetation extent with the PR-survey positions, to establish uni- and/or multivariate statistical relationships between malaria endemicity and the environmental factors that affect the distribution of the mosquito vectors responsible for malaria transmission. The basis for the estimation stems from the vector-transmitted nature of P.falciparum which is very sensitive to climatic factors.*

*The MAP utilized multiple sources of information including annual parasite incidence (API) data, malaria intervention coverage indicator data, parasite rate data, treatment-seeking data, satellite data to provide covariate raster surfaces, blood disorder data, mosquito bionomics and distribution data obtained from countries, WHO regional offices, localized surveys as well as expert opinions to arrive at the conclusive data.*

*MAP has gathered about 60,000 survey records from published sources that contain data on the infection status of nearly 5 million individuals. These are largely concentrated in Africa, with survey data in the other parts of the world not readily available.*

2. Provide summary tables (can go in SI) for which years STH prevalence estimates were available by country and how many prevalence surveys per country were available, as the sparsity of the data will influence your estimates. Ideally, something similar for the MAP estimates should be shown too.

*Response: As requested, we have provided in the supplementary table 2 a summary table showing the STH prevalence estimates by year and by country and the number of prevalence surveys available per country. A similar summary table has been provided for the MAP estimates in supplementary table 3.*

3. You are using estimates from MAP yet direct prevalence measurements from ESPEN. Why? Please explain how the methods for MAP and methods for STH are the same or differ in the prevalence kriging or estimation. If MAP data is not from prevalence surveys then discuss what the key dominating environmental features are of that data and how those environmental features feature into STH estimation (if at all). The introduction would have already given us an idea of the importance of each feature for each set of infections.

*Response: As explained in point 1 above, MAP used data points generated from malaria surveys for its estimation by combining these data points with GIS data To address the concern raised by the reviewer, we ensured that both malaria and STH datasets were on the same spatial resolution by calculating the population-weighted prevalence of malaria for each implementation unit using the population density data obtained from the WorldPop database. We have also provided additional information on the importance of each feature of the set of infections in the introduction.*

Reviewer #2:

4. The authors estimated the overall prevalence for each species over 18 years (i.e. 2000-2018). However, due to many control programs done in this period, the infection risk for each species changed a lot over the study period, temporal effect should not be ignored. As MAP provides estimate prevalence of *P. falciparum* for each year, and ESPEN provides STH survey data from 2000 to 2018, the data which the analysis based includes temporal information. Advanced methods should be used to take into account temporal effect.

*Response: We have revised the manuscript using the prevalence estimates of the STH infection for each year. Sartorius et al (2021)^1^ developed a multivariate spatiotemporal model for the three species of STH using Bayesian hierarchical framework. We used the estimates provided in Sartorius and colleagues’ paper to determine the hotspots of P.falciparum-STH co-infection. We have also taken the temporal effects of control programmes into consideration by revising the manuscript to reflect the transmission hotspots before and after the scale-up of the control programmes. Given that most of the control programmes were scaled up before 2003, we calculated the prevalence estimates before and after 2003. We also highlighted in supplementary figures 1-4 the spatial auto-correlation for P. falciparum and the co-infections with the three species of STH during these periods.*

5. The authors used the inverse distance weighted kriging methods to produce STH prevalence surface. However, they did not provide any further methodology on how to validate the model performance. For example, how to assess the accuracy of the method on estimating the prevalence where no survey data exist? In addition, as the prevalence surfaces were produced from spatial interpolation instead of real observation, methods and results for prediction uncertainty should be provided.

*Response: As explained in the response 4 above, we have revised our manuscript by using the spatiotemporal estimates, therefore the inverse distance kriging method is no longer used.*

6. In line 164, please explain what “spatially lagged variables” means.

*Response: We have revised the sentence as follows in lines 211-215: We used the bivariate Moran’s I scatter plot to show the degree to which the prevalence of a given variable at an IU is correlated with its neighbours for a different variable. The neighbours of the other variables is what we referred to as spatial lag of the other variables.*

7. In lines 165-167, the authors define high prevalence and low prevalence as values above and below the mean, respectively. What is the rationality for this definition? For example, if the prevalence for one species is very high across the study region, e.g., the prevalence of all areas is above 50%, then defining the areas with prevalence lower the mean level as “low prevalence” areas seems not reasonable. Besides, was the prevalence standardized before analysis? All prevalence should be above or equal to zero, however, the authors used the “above zero” and “below zero” in the definition.

*Response: We have clarified the definition of high and low prevalence in lines 216-228 of the revised manuscript, where we explained how we generated Moran’s I scatter plost using the z-scores and the z-scores enabled us to compare the prevalence estimates of any two variables, given that they might have different mean and standard deviation. Z-score value equal to zero was interpreted as the mean prevalence, above zero as higher than the mean prevalence and below zero as lower than the mean prevalence. Hence, on the scatter plots, all points with values above the mean (i.e. above zero) were IUs with higher than mean prevalence while all points with values below the mean (i.e. below zero) were IUs with lower than mean prevalence . We standardised the prevalence values using the z-score before analysis. We agree with the reviewer on their submission and we have revised the manuscript to reflect this accordingly.*

8. In lines 188-189, how to define the “high-risk clusters of *P. falciparum*, hookworm and *A.* *lumbricoides*”? Besides, the numbers of people in these clusters should be put in the result section instead in the methodology section.

*Response: Given that the information provides background information on the global estimates of the high risk clusters of individual and mixed infections involving P.falciparum and STH species and not direct outputs of the analysis of the datasets that we conducted for this paper, we feel that the sentence should not be in the result section. We have now moved this sentence to the Introduction section of the manuscript (in lines 147-151).*

Reviewer #2:

9. The authors provide results for co-infection of *P. falciparum* and each species of STH. Results for co-infection of malaria and any species of STH seems more meaningful for integrated control programs, as control methods for the three species of STH are similar. Such results should also be provided.

*Response: We thank the reviewer for their comment. We have provided in Figure 1 the prevalence of co-infection with P.falciparum and any of the STH species.*

10. The authors put the estimated prevalence of each species in the attachment. As these results are important, they should be put in the main text and more description of these results should be provided.

*Response: We have provided the estimated prevalence of each species in the main text of the manuscript in lines 239-324. We have also provided a detailed description of these results under the same section.*

11. In Figs 1A-1C, the prevalence below “-1” seems cut. What is the reason for this? Besides, how could prevalence below zero?

*Response: Thanks for your comment. We have revised this to highlight that the values presented on the scatter plots are not ‘prevalence values’ but z-scores.*

Reviewer #1: DISCUSSION

12. A major limitation of the study is the 5km resolution, especially for STHs.

*Response: We have revised the analysis of STH using a spatio-temporal estimates at implementation unit level. Therefore, we do not need to make prediction at 5km resolution any longer and the cluster analysis was also performed at implementation unit level.*

13. Information is needed on the effect of key ecological features in the estimations. Discuss gradients of infection prevalence for both malaria and hookworm as one moves away from water bodies; this will affect the interpolation.

*Response: We have provided the requested information in lines 118-130*

14. Rerun the analysis from 2003- onwards as this period is when MDA was substantially scaled up and discuss whether there is any heterogeneity pre 2003 versus post. Also, the breakdown of data pre-post MDA and its discussion is needed.

*Response: As requested, we have re-run the analysis from 2003 and illustrated the findings on pages 11,13,14 and 15 and discussed the findings before 2003 and from 2003-2018. We have also provided the breakdown of the data pre-post MDA in Figures 1,2,3, 4 and supplementary table 1. The narratives on these point estimate data were provided in lines 244-324.*

15. Discuss limitations of focusing on school-aged children, as hookworm at least is known to increase in prevalence/intensity with age.

*Response: We have discussed in the study limitations (lines 369-376) the focus on school age children and its implications on the interpretation of our findings.*

Editorial and Data Presentation Modifications

Reviewer #2: Attentions should be paid to the writing, as following:

16. In many places of the manuscript, species was not written italic, and there is no space between the first and the second words of the species. For example, errors in line 231.

*Response: We thank the reviewer for this observation. We have now written all species in italic and ensure there are spaces in between the first and second words of the species.*

17. Presentation of the figures should also be revised. For example, Fig 1 should be a figure instead of separating it into Fig 1A, Fig 1B and Fig 1C. Please follow the standards of the journal.

*Response: All figures have been revised in line with the journal’s guideline and have been presented as composite figures instead of fig 1A, fig 1B, etc.*

18. The positions of the titles of figures should also be revised according to the journal standards. For example, the positions of Fig 3A should be below the figure.

*Response: Thank you for this important comment. All figures have been revised according to journal style.*

19. Authors should be carefully check the typing errors. For example, in line 209, does “high prevalence of *P. falciparum* and high HW co-infection” mean “high prevalence of *P. falciparum* and HW co-infection”? Besides, if “HW” is the abbreviation of hookworm, it should be mentioned at the first time it appears.

*Response: We have carefully checked and rectified all typing errors in the manuscript. We have revised the sentences to make them clearer. We have also written HW in full before using it in abbreviated form*

20. In fig 2B, 3B and 4B, “probability” should be revised to “P value”, as it presents P value for statistical inference instead of the real probability of the events happening.

*Response: The results section have been re-written and all comments were taken into consideration during the process*

21. Fig 4A and Fig 4B seem in the wrong positions.

*Response:* The results section have been re-written and all new figures have been placed in their correct position.

Reviewer #1: I read this paper with great interest. It is well written and produces maps and estimates of spatial autocorrelation for STH and malaria coinfections on the African continent. More detail is needed on the methods. The purpose of the analysis must be made clearer, as the environmental factors that might lead to overlap in unclear (given that the sets of infections have vastly different transmission routes, and this was not discussed). The limitations of the analysis need to be better described.

*Response: We thank the reviewer for their kind feedback. We have provided additional details in the methods section. In lines 118-128, we have also described the roles of environmental factors that might contribute to the overlap of malaria and STH infections. Also, we have expanded on the limitations of this analysis.*

ABSTRACT

22. Lines 42-43: *P. falciparum* is repeated.

*Response: We have revised the sentence to make it clearer*

23. The abstract needs to directly state the lowest resolution (km) where coinfection estimation was possible. Also, clarify in the abstract whether STH prevalence was georeferenced when estimated or if the implementation unit for STH prevalence was used and then a point estimate was created in the middle of that implementation unit. And, directly state the age range at which these coinfection estimates are applicable.

*Response: We have revised the abstract to address the comment of the reviewer in lines 33-40 as follows: Although, MAP provides datasets on the estimated prevalence of P. falciparum at 5km x 5km fine-scale resolution, we calculated the population-weighted prevalence of malaria for each implementation unit to ensure that both malaria and STH datasets were on the same spatial resolution. We incorporated survey data from 5,935 implementation units for STH prevalence and designated the prevalence estimates before 2003 as pre-intervention while those that occurred from 2003 to 2018 as post-intervention. We used the bivariate local indicator of spatial association (LISA analysis) to explore potential co-clustering of both diseases at the implementation unit levels among children aged 2-10 years for P. falciparum and 5-14 years for STH.*

INTRO

24. Throughout the introduction, provide prevalence estimates with their relevant administrative unit.

*Response: We have provided prevalence estimates with their relevant administrative unit in the introduction section of the manuscript.*

25. Given the vastly different transmission routes and the localized influence of soil conditions on STH infections, please expand the introduction to provide a background of exactly what environmental factors, including their directionality of influence, overlap between the two sets of infections. Reporting co-endemicity by country is too vague. Literature on the epidemiology of the two infections is needed and past empirical findings should be noted.

*Response: We have revised the introduction by providing information on the roles of environmental factors shaping the co-infection. We have also added literature on the epidemiology and past empirical findings on the co-infection.*

26. Lines 107-109: Clarify whether these estimates are at the country level.

*Response: We have clarified the estimates by adding ‘at country level’* *to the sentence*

27. Reviewer #2: Using data from MAP and ESPEN and methods of spatial cluster analysis, the authors identified high co-infection areas of malaria and each species of STH. The research topic of the manuscript is meaningful for integrated control programs for malaria and STHs. However, there are several important points regarding to the methods and the results needed to address, as described in the above “Methods” and “Results”. Besides, the manuscript seems not well organized, and concerns about the title and the writing should also be paid attention.

*Response: We have revised the methods and results sections significantly to address the concerns raised by the reviewer. We have also re-organised the manuscript, revised the title and the writing to make the manuscript more readable.*

28. Regarding to the title, the “burden” in the title seems not proper as the manuscript mainly provided results for high co-infection areas, instead of the estimation of disease burden due to co-infection. For writing concerns, please refer to the above “Editorial and Data Presentation Modifications”.

*Response: We have revised the title to read as follows: Prevalence and distribution pattern of malaria and soil-transmitted helminth co-infection in sub-Saharan Africa, 2000-2018: a geospatial analysis*

***Reference***

1. *Sartorius, B., Cano, J., Simpson, H., Tusting, L. S., Marczak, L. B., Miller-Petrie, M. K., ... & Pullan, R. L. (2021). Prevalence and intensity of soil-transmitted helminth infections of children in sub-Saharan Africa, 2000–18: a geospatial analysis. The Lancet Global health, 9(1), e52-e60.*
