# Supplementary material for: Prevalence and distribution pattern of malaria and soil-transmitted helminth co-endemicity in sub-Saharan Africa, 2000–2018: A geospatial analysis
Source: PLoS Negl Trop Dis. 2022 Sep 30;16(9):e0010321. doi: 10.1371/journal.pntd.0010321 (PMC9555675; doi:10.1371/journal.pntd.0010321)
Supplement: Attachment — Submitted filename: RESPONSE SHEET_PNTD-D-22-00324R1.doc [file pntd.0010321.s009.doc]

30 August 2022

Dear Editor,

Revision of manuscript PNTD-D-22-00324R1: Prevalence and distribution pattern of malaria and soil-transmitted helminth co-endemicity in sub-Saharan Africa, 2000-2018: a geospatial analysis.

Thank you for sharing the editorial and reviewers’ comments on the above named manuscript. We have carefully taken the reviewers’ comments and suggestions into account in the revision of the manuscript. We describe below how we have responded to specific comments. We have addressed the concerns from the reviewers; and hope this paper is now acceptable for publication in your journal.

Yours sincerely,

Muhammed Afolabi

On behalf of all co-authors

**Reviewers' comments:**

**Reviewer #1: The authors have addressed my requests and greatly improved the manuscript; minor changes are needed for the introduction and terminology before acceptance.**

**Given that the authors are not looking at the same set of people for malaria and STH nor are they looking at the same age range, using the term co-infection may be misleading. Instead, the manuscript should be revised to clearly note geographical co-endemicity. Co-distributions might also be appropriate as already used in the lay summary. Choose one terminology and use throughout the manuscript.**

***Response: We thank the reviewer or their kind feedback. As requested, we have replaced ‘co-infection’ with ‘co-endemicity’ throughout the manuscript.***

**The additional description of shared environmental or other drivers between malaria and STHs is poorly written without clear examples. Clearly state exactly what climate-related factors predicted both malaria and STHs. Also, note what ‘immune modulation’ is responsible for mixed infections; there is not an interaction between malaria and STH that affects susceptibility to infections, so this sentence is misleading. Clarify what socioeconomic factors affect both diseases. There is still no discussion here of the vastly different transmission routes or differences in climatic factors.**

***Response: Thank you for raising these important points. The importance of climatic and socio-economic factors that have an impact on both groups of infections is now discussed in lines 126-133,***

***‘The climatic factors that predicted both malaria and STH and clarified socio-economic factors affecting both diseases: Changes in temperature, humidity, rainfall, and other climatic conditions have an impact on key factors that shape the transmission of malaria, such as the mosquito lifespan and the development of malaria parasites in the vector. [11] Similarly, the interactions between the climatic parameters affect rates of desiccation and mortality for eggs of A. lumbricoides and T. trichiura, and hookworm egg hatching success, thereby affecting the overall survival of the STH parasites and development of infective larval stages. In addition, socio-economic conditions such as agricultural practices and other human behaviours also alter ecological landscape, soil larval loads, vector survival, food security and overall human health and wellbeing, thereby providing favourable interface for transmission and co-distribution of malaria and STH.’***

***We have also revised the sentence on immune modulation in lines 133-135 to read as follows:*** ***The roles of biological factors driven by immune modulation were also reported to be responsible for the co-existence of the mixed infections.***

**Reviewer #2: The authors revised the manuscript according to editorial and reviewers’ comments. It seems much better compared to the original one. Only one comment: The authors used Sartorius et al’s estimates for STH infection instead of the original kriging method. Are these estimates also based on ESPEN portal? Please clarify it.**

***Response: We confirm that the estimates for STH infection used by Sartorius et al are based on the data obtained from the ESPEN portal.***
